# Growth, physicochemical properties, and morphogenesis of Chinese wild-type PRV Fa and its gene-deleted mutant strain PRV SA215

**DOI:** 10.1186/1743-422X-8-272

**Published:** 2011-06-04

**Authors:** Ling Zhu, Yue Yi, Zhiwen Xu, Lu Cheng, Shanhu Tang, Wanzhu Guo

**Affiliations:** 1 Animal Biotechnology Center of Sichuan Agricultural University, Yaan, Sichuan (625014), PR China; 2 ChengDu University of Technology,ChengDu,Sichuan,PR China

**Keywords:** pseudorabies virus, gene-deleted mutant strain, growth and physicochemical properties, morphogenesis

## Abstract

**Background:**

PRV Fa is common in China and causes most of the pseudorabies in the pig industry. A PRV SA215 strain with deleted gE, gI, and TK genes was constructed to develop a commercial attenuated live vaccine. However, the physicochemical properties, growth pattern, penetration kinetics, and morphogenesis of the PRV SA215 and its parental PRV Fa strain are unclear.

**Results:**

A series of experiments were conducted to characterize both strains and provide more information. PRV Fa and PRV SA215 were found to have similar penetration patterns, with about 5 min half-time of penetration. The SA215 strain exhibited a slight delay in entry compared with PRV Fa. In the one-step growth test, the titers of the SA215 strain were first detected at 8 h, rapidly increased, and peaked at 12 h. A plateau was formed between 12-36 h of culturing. PRV SA215 showed delayed replication and approximately 10-30-fold lower titers during 0-16 h of culturing compared with the PRV-Fa strain. After 16 h, the PRV Fa titers dramatically decreased, whereas those of PRV SA215 were prolonged to 36 h and reached a titer value equal to that of PRV Fa and then decreased. Both strains were sensitive to both heat and acid-alkali treatments; however, PRV Fa was relatively more stable to heat treatment than PRV SA215. Both strains could propagate in the cultures with pH values from 5.0 to 9.0. Cultures with pH below 3.0 or above 11.0 were fatal to both strains. Both strains had considerable resistance to freeze-thawing treatments. Morphogenetic investigations showed that typical phases in the maturation pathway were observed in the PRV Fa-infected PK15 cells, whereas secondary envelopment was not observed in the PRV SA215 strain. Instead, capsid aggregations with concomitants of electrodense materials were observed.

**Conclusions:**

These results suggest that PRV SA215 is a promising strain for vaccine development

## Background

The *pseudorabies virus *(PRV) is a member of the *Alphaherpesvirinae *subfamily in the family *Herpesviridae*. It is the causative agent of Aujeszky's disease [1]. The disease caused by PRV was first observed in cattle and described as "mad itch" [2]. Swine is the primary host and reservoir of this virus. PRV is able to infect most mammals and some avian species. In young piglets, as well as in the other susceptible species, PRV infection is often fatal, and animals die from central nervous system disorders, whereas older infected pigs usually primarily develop respiratory symptoms. Similar to other alpha herpesviruses, PRV infection is a life-long latent infection of the peripheral nervous system. These latently infected pigs can serve as sources of renewed infection when the latent virus genome spontaneously reactivates and infectious viruses are produced. In pregnant sows, PRV infections may cause the death of fetuses and/or abortion. Thus, PRV is a pathogen with major agricultural effects and economic importance [3].

Pigs are commonly vaccinated with attenuated live PRV vaccines [4] to control the disease. These vaccine strains are attenuated by inactivation of one or more genes that encode nonessential proteins. Studies found that glycoprotein gE is dispensable for viral growth in animals or in the cultures [5,6]; however, it is necessary for virulence and virion replication [7,8], and plays an important role in virion spread from cell to cell. A virus with only a deletion of the gE gene is capable of replicating *in vivo *and *in vitro *with considerable virulence. Thus, most of the commercialized attenuated live vaccines are constructed based on gE deletion combined with one or more of other gene deletions such as TK [9]. However, this kind of vaccine has an attenuated virulence and restricted infectivity. Inactivation of viral genes was reported to often result in reduced replication capacity. The highly virulent strain NIA-3, the intermediately virulent strain 2.4N3A, and the non-virulent strain Bartha were compared in a study of the pathogenicity of different PRV strains. The results showed that the degree of virulence is directly related to the ability of these viruses to replicate in the nasal epithelium [10]. Reduced replication lowers the viral antigen supply and may impair the immunogenicity of the vaccine.

A gene deficient strain SA215 (gE-/gI-/TK-) was constructed in our laboratory based on a Chinese PRV Fa strain [11]. The PRV Fa strain is the earliest isolated typical strain that caused the prevalence of pseudorabies in China and caused large economic losses in the pig industry. The biological and physicochemical properties and morphogenesis of both strains are still unclear. Preliminary studies have shown that the PRV SA215 strain has biologically secure properties with high immunogenicity and long-term immunity, and could be developed as a commercial attenuated live vaccine. However, its growth, physicochemical properties, and morphogenesis during multiplication have not been investigated. In the current paper, the results of recent studies on the physicochemical properties and growth pattern, penetration kinetics and morphogenesis are presented to provide more information about the Chinese typical wild-type PRV Fa strain and its progeny, the PRV SA215 strain.

## Materials and methods

### Viruses and cells

The PRV SA215 strain was constructed by our center (Animal Biotechnology Center of Sichuan Agricultural University) based on the Chinese PRV Fa strain. PRV SA215 carries gE, gI, and TK gene deletions. The strongly virulent wild-type PRV Fa strain was obtained from the Institute of China Veterinary Medicine Inspection. PK15 cells and Vero cells were purchased from CCTCC. DMEM, trypsin, and fetal calf serum were purchased from GIBCO Inc. Methylcellulose was obtained from Sigma Inc. Vero cells were used in the vaccine strain propagation, plaque formation test, isolation and titration of the PRV virus in nasal discharges, and sera neutralization assays.

### PRV Passaging

Vero cells were cultured in DMEM at 37°C and 5% CO_2_. The nutrient solution contained 10% fetal calf serum and 100 U/ml of penicillin and streptomycin. The growth liquids were poured out when a dense cell monolayer was formed in the culture flasks. The cell monolayers were washed twice with calcium- and magnesium-free water. 1-2 drops trypsin were added into each flask to trypsinize the cells. The cells were split 1:2 for subsequent cultures.

### Plaque formation test

The test was carried out to measure virus titration following our regular laboratory protocol. Briefly, the Vero cells were grown on 6-well plates until the appearance of cell monolayers. The virus samples were diluted 10 times and the samples with appropriate viral titers were selected for inoculation, and 0.1 ml of this sample was added into each well. The subject culture samples in the plates were gently shaken to mix the samples well and allowed to adsorb for 1 h at 37°C. A solution of 1% methylcellulose was added to the wells to cover the culture medium. After the tested samples were cultured for 3-5 days at 37°C in a CO_2_-controlled incubator, the methylcellulose was aspirated and 1 ml of formalin crystal purple staining solution was added to fix and stain the samples for 20 min. The staining solution was removed by washing with tap water. The plaques were counted and the PFU were calculated based on the volumes of the original samples.

### Penetration kinetics

The penetration kinetics of both strains was assayed using low-pH inactivation of the extracellular virus based on the method described by Mettenleiter [12]. Briefly, PRV Fa and PRV SA215 were inoculated on the PK15 monolayer cells. The input virus amount was ca. 200 PFU per well in a six-well tissue culture plate. After adsorption for 1 h at 4°C, the inoculants were removed and the cells were covered with nutrient solution at 37°C to facilitate virus penetration. A lower pH solution (pH 3.0, 40 mM citric acid-10 mM KCl-135 mM NaCl) was added to the wells for 2 min to inactivate the extracellular viruses after 0, 5, 10, 20, and 40 min of penetration. The lower pH-treated cell monolayers were washed twice and covered with methylcellulose solution. After culturing for 2-3 days, the cells were fixed and stained. The plaques were counted and the penetration percentages were calculated by comparing with the number of plaques formed in the PBS-treated control.

### One-step growth analysis

One-step growth analysis was performed according to the method described by Klupp et al. [13]. The PK15 monolayer cells were infected with PRV Fa and PRV SA215 at 0.005 multiplicity of infection (MOI) in each well of a six-well tissue culture plate. The viruses were allowed to adsorb for 1 h at 4°C. The extra viruses were removed and the infected cells were covered with nutrient solution at 37°C. After 90 min of culture and penetration, the extracellular virus was inactivated with a low pH solution. The maintenance growth solution for the cells was then switched. The cell supernates and their mixtures were collected at 0, 4, 8, 12, 16, 20, 24, 28, 32, 36, 40, 44, and 48 h. The cells were frozen at -70°C and lysed by rapidly thawing at 37°C. Virion amount in different portions was determined by the plaque formation test.

### Assays for the physicochemical properties of the virus

Conventional methods were applied in the investigation of physicochemical properties, which included testing PRV heat-resistance, sensitivity to pH, and stability to freeze-thawing treatment. (1) Both the PRV Fa and PRV SA215 strains were heat-treated to test their heat-resistance. Flasks containing either PRV Fa or PRV SA215 were incubated at 56°C and the titers were measured after incubation for 0, 15, 30, 45, or 60 min. (2) PRV sensitivity to acids and alkali were also investigated by adjusting the culture medium pH to 3.0, 4.0, 5.0, 6.0, 7.0, 8.0, 9.0, 10.0, and 11.0 with 0.1 M HCl/NaOH solutions. All the cultures were incubated at 37°C for 1 h, and the pH values were then adjusted back to 7.0. The plaques were measured to determine the virus titers and sensitivity to pH. The titers were evaluated by calculating the acid and alkali amounts added based on the original sample volumes. (3) In the freeze thaw treatment, the PRV viruses were repeatedly frozen below 20°C and thawed at 37°C 1 to 5 times to determine the effects of freeze-thawing on PRV multiplication, as indicated by titers. All samples were assayed for titers after the freeze-thaw treatment.

### EM of PRV

The procedure described by Granzow et al. (1997) [14], modified for routine electron microscopy (EM) observation, was used. The PK15 cell monolayers were infected with PRV Fa or PRV SA215 at a MOI of 10. Both non-infected and infected cell cultures in Petri dishes were fixed at different times (12, 16, 20, and 24 h post-infection) after infection for 60 min with 2.5% glutaraldehyde buffered in 0.1 M Na-cacodylate (pH 7.2, 300 mOsmol. They were then scraped off the plate and pelleted by low-speed centrifugation and embedded in LMP agarose (GIBCO Inc). Small pieces were postfixed in 1.0% aqueous OsO_4 _(company, city, country) and stained with uranyl acetate. After stepwise dehydration in ethanol, the cells were cleared in propylene oxide, embedded in agarose, and polymerized at 59.8°C for 4 days. Ultrathin sections, counterstained with uranyl acetate and lead salts, were examined for morphogenesis using a transmission electron microscope (H600 Japan).

## Results

### Penetration kinetics

**Figure 1:**
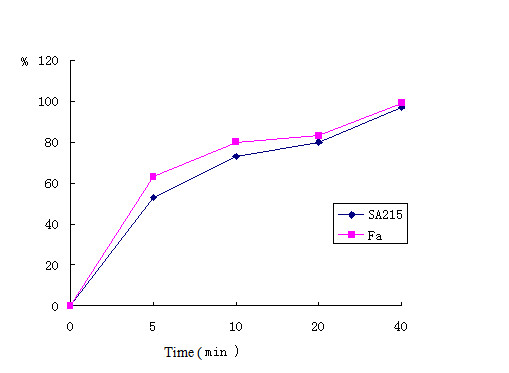
**Penetration kinetics of PRV Fa and PRV SA215**. The penetration kinetics of PRV Fa and PRV SA215 into Vero cells was analyzed by low-pH inactivation. Indicated is the percentage of PFU that was resistant against treatment with pH 3.0 citrate buffer compared with the control treated with phosphate-buffered saline at different times after the temperature shift. Averages from three independent experiments are indicated.

The entry kinetics of PRV Fa and PRV SA215 were determined using low-pH inactivation of the remaining extracellular viruses to analyze viral penetration. As shown in Figure 1, PRV Fa and PRV SA215 showed similar penetration patterns and about 5 min half-time penetration, although SA215 exhibited a slight delay in entry compared with PRV Fa. These results indicate that both strains have similar penetration ability, and the penetration was not arrested in the constructed strain PRV SA215.

### PRV one-step growth kinetics

**Figure 2:**
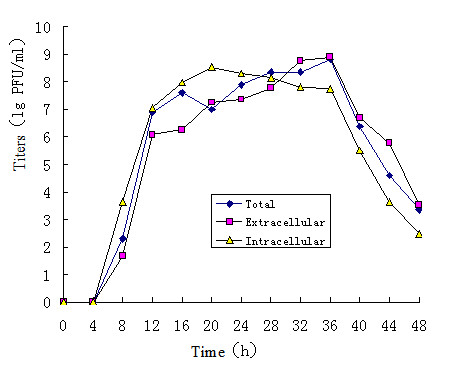
**One-step growth kinetics of PRV SA215**. Vero cells were infected with PRV SA215 at 5 MOI. Cells, supernates, and cell-supernate mixtures were harvested at the indicated time points and titrated on Vero cells. Averages and standard deviations from three independent experiments are shown.

**Figure 3:**
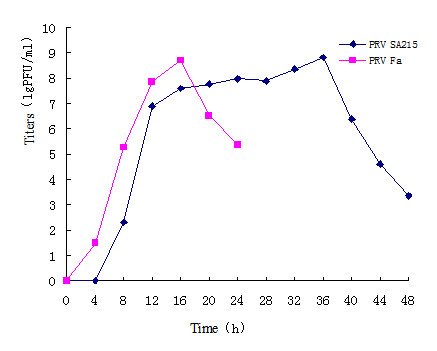
**One-step growth kinetics of PRV SA215 and Fa**. Vero cells were infected with PRV SA215 at 5 MOI. Cells were harvested at the indicated time points and titrated on Vero cells. Averages and standard deviations from three independent experiments are shown.

Vero cells were infected with the wild-type PRV Fa and the constructed PRV SA215 strain at 5 MOI to assay the replicative abilities of the two viruses. The cells, supernates, and their mixtures were collected for titer investigation. At various time points, the virus titers of the Vero cells were determined. The results are shown in Figures 2 and 3. In the current study, the titers in the SA215 strain cultures were determined at 8 h. After 8 h, the virion titers rapidly increased and maintained a high plateau during the 12-36 h culture period. The corresponding pathologic changes were cell aggregation, fusion, and formation of plaques at approximately 80% CPE. PRV SA215 replicated with a delay and approximately 10-30-fold lower titers during the 0-16 h culture compared with the PRV-Fa strain. After 16 h, the PRV Fa titers dramatically decreased, whereas those of PRV SA215 slowly crept to 36 h and reached a titer value similar to PRV Fa, before rapidly decreasing. In addition, PRV Fa was observed to cause cell desquamation and crashing in a relative short time (28 h).

### Physicochemical properties of the virus

**Figure 4:**
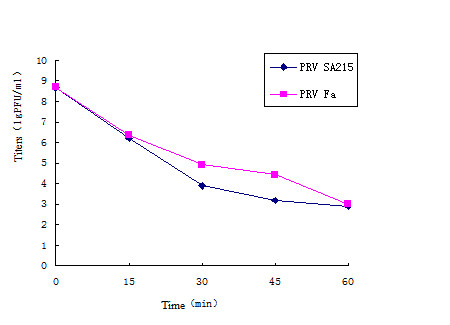
**Titers determined at different time points of heat treatment**. Both strains were cultured under 65°C for 0, 15, 30, 45, or 60 min and titers were determined at each time point. Averages and standard deviations from three independent experiments are shown.

**Figure 5:**
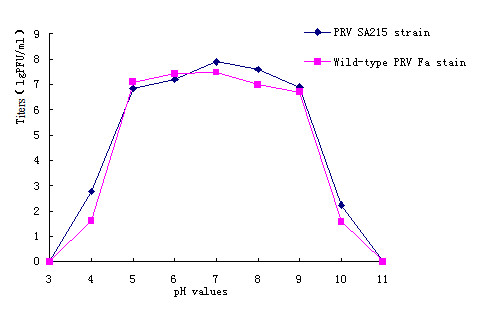
**Titers in cultures with varied pH**. The viruses were cultured at varying medium pH. The titers were assayed. Averages and standard deviations from three independent experiments are shown.

**Figure 6:**
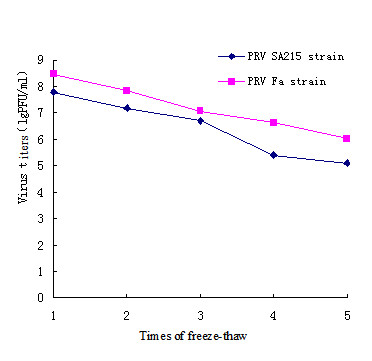
**PRV titers influenced by freeze-thawing treatments**. The PRV virus was treated with different freeze-thawing durations and cultured at 37°C for 1 h. Titers were determined in each treatment. Averages plotted and standard deviations from three independent experiments are shown.

The physicochemical properties of the wild-type PRV Fa and the constructed PRV SA215 were compared in the current study. The results from the heat-resistance test, pH sensitivity, and stability to freeze-thawing treatment are shown in Figures 4, 5 and 6.

The heat treatment of PRV SA215 and the wild-type PRV Fa virus at 65°C at varying durations resulted in a significant reduction in the titers in both strains, with approximately 2 titers reduced in each 15 min slice during the heat treatment (Figure 4). From heat treatment time 0 to 60 min, the titers for both strains decreased from approximately 9 to 3 lgPFU/ml; however, the PRV Fa strain showed relatively higher titers at different time points compared with PRV SA215 during the heat-resistance test.

In different acid-alkali environments, both strains showed similar sensitivity patterns (Figure 5). Both PRV SA215 and PRV Fa were relatively stable in the cultures with pH values of 5.0 to 9.0, and showed high titers of around 7 lgPFU/ml. The virion titers rapidly decreased when the culture pH was changed to less than 5.0 or higher than 9.0. No detectable titers were observed and all viruses were inactivated when the culture medium pH was adjusted to 3.0 or 11.0.

The effect of repeated freeze-thawing on the titer measurements of the two virus strains are shown in Figure 6. The freeze-thawing treatment decreased the titer values. From 1 to 5 cycles of freeze-thawing treatment, the titers of PRV Fa and PRV SA215 decreased from 8.5 to 6.0 lgPFU/ml and 8.0 to 5.0 lgPFU/ml, respectively.

### Morphogenesis of PRV SA215

The morphogenesis of both the PRV SA215 strain and its parental strain PRV Fa on PK15 cells were investigated and compared under an electron microscope to test the effects of the PRV SA215 strain on the replication in infected cells.

**Figure 7:**
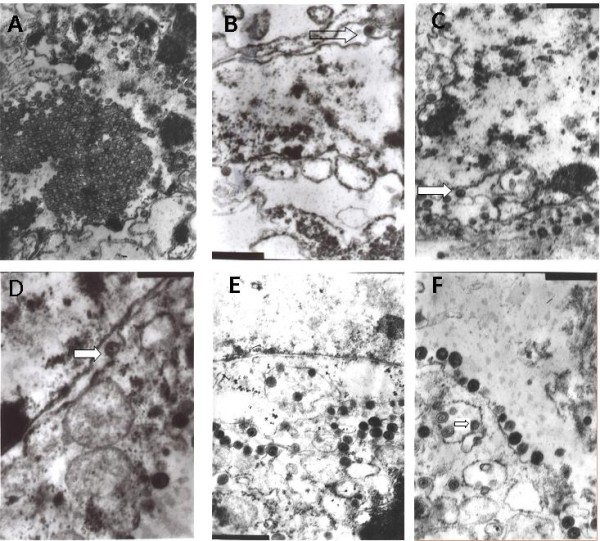
**Morphogenesis of the wild-type PRV Fa strain**. Formation of capsids in the PK15 cell nucleus and a large number of nucleocapsids aggregated and arrayed as pseudocrystals were observed under 25,000 × amplification (A). The gap in the nuclear membrane was distended and a nucleocapsid was budding through the inner nuclear membrane to acquire a primary envelope indicated by the arrow (B, 30,000×). Virions in the envelope and numerous nucleocapsids scattered in the nucleus were observed (C, 25,000×). Fusion of a primary envelope with the outer nuclear membrane, and nucleocapsids released into the cytoplasm, and loss of a primary envelope; the arrow points to a budding process (D, 20,000×). A secondary envelope was acquired in the trans-Golgi area of the perinuclear cisterna (E, 25,000×). Newly produced viral progeny was released to the cell surface through the fusion of a transport vesicle and the cytoplasmic membrane via exocytosis (F, 30,000×).

A number of virions were found after the PK15 cells were infected with the PRV Fa strain. A typical herpesvirus nucleocapsid morphogenesis pathway in intranuclear maturation was identifiable, including nucleocapsid assembly (Figure 7A), formation of a secondary envelope from transportation via the inner nuclear membrane (Figures 7B and 7C), loss of the primary envelope and release of the nucleocapsid into the cytoplasm in the outer nuclear membrane (Figure 7D), formation of encapsulated vesicles and envelopes in the trans-Golgi area (Figures 7E and 7F), and the release of mature virions into the cytoplasm via transport vesicles (Figure 7F).

**Figure 8:**
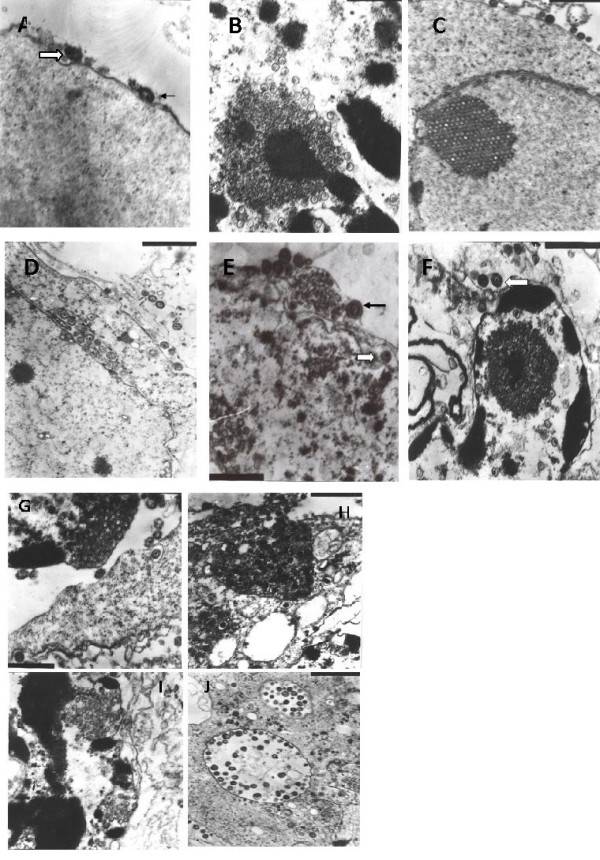
**Morphogenesis of the constructed PRV SA215 strain (A to J)**. Virion adsorption and penetration (A, 35,000×) and formation and release of nucleocapsids into the cytoplasm (B, 30,000×) were observed. A large number of nucleocapsids aggregated and arrayed as pseudocrystals in the cell nucleus with insignificant enlargement of the nuclear membrane, and virion budding into the nuclear membrane were seen (C, 20,000×). Nucleocapsids budding through the inner membrane to obtain a primary envelope and budding out from the outer nuclear membrane to gain a secondary envelope, and loss of a primary envelope in the endoplasmic reticulum (D, 20,000×). Nucleocapsid release through the nuclear membrane and virions wrapped with tubular vesicles (E, 30,000×). Cytoplasmic nucleocapsids gaining envelopes through budding into the vesicles (F, 30,000×). Virion release (G, 30,000×). A large number of non-envelope capsids aggregated with electron-dense concomitant materials were found in the cytoplasm (H, 20,000×). Numerous nucleocapsids amassed in the inner or inside the nuclear membrane (I, 25,000×). Several envelopes from the nucleocapsids were observed in the nucleus (J, 15,000×)

In the current study, virion adsorption, nucleocapsid formation, and DNA encapsulation were not influenced by gene deletions (Figures 8A, B and 8C) in the PRV SA215 strain. Nucleocapsids acquired primary envelopes by budding through the inner nuclear membrane, and were released into the cytoplasm through nuclear membrane fusion and loss of the primary envelope (Figures 8D and 8E). Nucleocapsids obtaining secondary envelopes by budding into the vesicles in the trans-Golgi area (Figure 8F) and the mature virions being released outside the vesicle via transport vesicles through exocytosis were observed (Figure 8G). Some abnormal morphologic changes were also observed, such as the inclusion bodies of non-envelope capsids with concomitants of electrodense materials derived from the coating membrane proteins (Figure 8H), and arrest in the budding through the inner nuclear membrane, resulting in a mass of nucleocapsids in the inner or inside the nuclear membrane (Figure 8I). Amazingly, a large number of vesicles from the nucleocapsids in the nucleus were also observed (Figure 8J). These results indicate that the formation of secondary envelopes and virion exocytosis could be observed under EM after the PK15 cells were infected with PRV SA215. However, electrodense cytoplasmic materials were found, as well as capsids, which demonstrate that budding in the nuclear membrane and envelope formation were impaired or delayed to some extent.

## Discussion

The initiation of PRV adsorption in target cells is reportedly associated with the interaction of gC and acetyl heparin, with the secondary adsorption mediated by gD [15]. The adsorption is followed by fusion of the viral envelope with the cytoplasmic membrane. Glycoproteins gB, gD, gH, and possibly gL, are associated with and essential for the fusion process [16]. The results of the current study indicate that the adsorption and penetration of the gE-/gI-/TK-PRV SA215 strain were not impaired, which infers that gE, gI, and TK were not essential for PRV adsorption and penetration. PRV SA215 showed a postponed appearance of detectable titers and slowed growth compared with the parental strain PRV Fa. However, equal titers in the SA215 strain and in its parental strain Fa were observed. SA215 was also found capable of maintaining high titers for a much longer time than PRV Fa. These results are in agreement with the expected results of the functional deficiency of the gE, gI, and TK genes. PRV uses two methods to spread virions in the infection: a direct cell to cell spreading, and releasing virions for adsorption by uninfected cells. The first method is mediated by the glycoprotein gE, whereas the latter requires glycoprotein gC involvement. Although the gE gene-deleted mutant strain lacks the capability to directly spread virions from cell to cell, this strain can use the second mechanism to be adsorbed by uninfected host cells and then infect and proliferate in the cells to produce titers equal to that of the wild-type PRV [17]. In PRV infection, gI exerts its functions by forming a complex with gE. Mutant strains with either gI, gE, or both genes deleted were reported to have similar biological properties [18]. All these results demonstrate that SA215 can easily grow and proliferate in cells, and can be used as a commercial vaccine strain.

In the current study, the physicochemical properties of PRV SA215 and its parental strain PRV Fa were investigated. The results showed that both strains have comparable features. Both strains were sensitive to heat inactivation. Prolonged heating at 65°C could kill all of the viruses. These strains were also sensitive to culture pH. Although they demonstrated relative stability to pH and retained high viral titers in the culture medium with pH values ranging from 5.0 to 9.0, their replications were inhibited when the culture medium pH were beyond 5.0 and 9.0. Furthermore, pH 3.0 and 11.0 were fatal to both strains. PRV Fa and PRV SA215 were relatively stable under the freeze-thaw treatment and showed considerable resistance to repeated freezing and thawing treatments. Interestingly, extending the vaccine shelf life and storage time based on these results is possible if the SA215 strain was used for vaccine production.

Generally, PRV virions are assembled stepwise in the host cell. Newly replicated DNA is encapsidated into nucleocapsids in the cell nucleus. These nucleocapsids are then translocated into the cytoplasm by envelopment and de-envelopment. In the cytoplasm, a secondary envelope is acquired by the budding of the nucleocapsids into the trans-Golgi vesicles, which are then transported to the cell surface [19]. In the current study, similar results were observed in PRV Fa-infected cells. All the typical maturation phases in morphogenesis were observed in the ultrathin sections of PRV Fa-infected pK15 cells under EM. Compared with the wild-type PRV Fa strain, virion adsorption, penetration, formation of capsids and DNA capsulization in nucleus, primary envelope formation by budding through the inner nuclear membrane and de-envelopment by fusion with the outer nuclear membrane, nucleocapsid release to the cytoplasm, secondary envelopment through nucleocapsid budding into the vesicles in the trans-Golgi area, and the release of mature virions to the cell surface during PRV SA215 morphogenesis were observed. In addition, some atypical morphologic changes in some parts of the infected cells were also observed. Numerous nucleocapsids amassed in the inner side and in the inside of the nuclear membranes, and the release of nucleocapsids was impaired. Normal primary envelopment during budding through the inner membrane was not observed. Interestingly, the amassed non-enveloped capsids with concomitants of electrodense materials from the coating membrane proteins in the cytoplasm were also observed. Brack et al. [20,21] reported numerous electrodense materials in the cytoplasm of cells infected with gE- and gI-deleted PRV strains; however, these authors did not observe capsids. In their studies, PRV morphogenesis was not affected by double gene deletions, and secondary envelopment and exocytosed virions were observed. The authors concluded that this phenotypic change was attributed to the loss of the interaction between the cytoplasmic tail of gE and the special capsid components.

## Conclusions

In the current study, the physicochemical properties of PRV SA215 and its parental strain PRV Fa were investigated. All these results suggest that PRV SA215 is a promising strain for vaccine development.

## Competing interests

The authors declare that they have no competing interests.

## Authors' contributions

LZ, YY and ZWX participated in the design and carried out the majority of the experiments in the study and drafted the manuscript.WZG have critically revised the manuscript and the experimental design. LC, SHT helped to draft the manuscript. All authors read and approved the final manuscript.
